# Yingyangbao Reduced Anemia among Infants and Young Children Aged 6–23 Months When Delivered through a Large-Scale Nutrition Improvement Program for Children in Poor Areas in China from 2015 to 2020

**DOI:** 10.3390/nu15112634

**Published:** 2023-06-05

**Authors:** Lijuan Wang, Junsheng Huo, Yanli Wei, Yanbin Tang, Jing Sun, Jian Huang

**Affiliations:** Key Laboratory of Trace Element Nutrition of National Health Commission, National Institute for Nutrition and Health, Chinese Center for Disease Control and Prevention, Beijing 100050, China; wanglj@ninh.chinacdc.cn (L.W.); huojs@ninh.chinacdc.cn (J.H.); weiyl@ninh.chinacdc.cn (Y.W.); tangyb@ninh.chinacdc.cn (Y.T.)

**Keywords:** Yingyangbao, hemoglobin, anemia, infant

## Abstract

The purpose of this study was to assess the effectiveness of intervention with Yingyangbao (YYB) on hemoglobin (Hb) and anemia status among infants and young children (IYC) aged 6–23 months (mo) through a large-scale Nutrition Improvement Program for Children in Poor Areas (NIPCPA) in China from 2015 to 2020. Five rounds of cross-sectional surveys were conducted using a stratified and multi-stage probability proportional to size sampling of IYC in 2015, 2017, 2018, 2019 and 2020. Multivariable regression analyses were fitted to determine the effectiveness of the YYB intervention on Hb and anemia, respectively. A total of 36,325, 40,027, 43,831, 44,375 and 46,050 IYC aged 6–23 mo were included, and the prevalence of anemia was 29.7%, 26.9%, 24.1%, 21.2% and 18.1% in 2015, 2017, 2018, 2019 and 2020, respectively. Compared with the results in 2015, the Hb concentrations significantly improved and anemia prevalence significantly decreased among IYC in 2017, 2018, 2019, and 2020 (*p* < 0.001). Regression analysis showed that higher YYB consumption was significantly associated with the increment in Hb concentration and reduction in anemia stratified by age group (*p* < 0.001). The most significant increment in Hb concentration of 2.189 mg/L and the most significant reduction in odds of anemia were observed among IYC aged 12–17 mo who consumed 270 to 359 sachets of YYB (OR: 0.671; 95% CI: 0.627, 0.719; *p* < 0.001). This study suggests that YYB intervention is a successful public health strategy for reducing the risk of anemia among IYC when delivered through a large-scale NIPCPA in China. It is necessary to continue to advance the program and increase the adherence of YYB.

## 1. Introduction

Anemia is a common public health problem that affects approximately 2 billion people, with serious adverse effects on public health and socioeconomic development. Children under 5 years of age are one of the most important vulnerable populations to anemia, especially in the first two years of life. The World Health Organization (WHO) estimates that 46% of children under 2 years of age suffer from iron deficiency anemia, especially in infants [1]. Iron deficiency in children is about two to three times more common than iron deficiency anemia due to rapid growth and inadequate dietary practices. IYC are vulnerable to suffering from iron deficiency, and anemia can occur if iron deficiency further progresses [2]. Iron deficiency anemia and iron deficiency affect the physical growth and mental development of children, increase child mortality and morbidity, cause reduced work capacity in adulthood and affect economic growth in high-risk areas [3,4,5].

In order to meet the physiological needs of IYC from 6 to 23 months (mo) of age, they also need to be provided with high-quality complementary foods other than breast milk. Internationally, home fortification has been widely used to improve the quality of complementary foods for IYC, and China has also developed Yingyangbao (YYB), which is a single-dose sachet of powder containing high-quality protein and micronutrients that can be mixed into any semisolid food at home or at the point-of-use to enrich the food with protein, alongside essential vitamins and minerals. In 2012, the Nutrition Improvement Pilot Program for Children in Poor Areas was launched by the Chinese government. The first 270,000 IYC aged 6 to 23 mo in 100 contiguous poor counties in central and western provinces were provided free YYB and promotional materials. In 2013, the project was renamed the Nutrition Improvement Program for Children in Poor Areas (NIPCPA) and was expanded to 300 counties in 21 provinces in contiguous areas of extreme poverty, benefiting 830,000 IYC. In 2014, the program coverage increased to 341 counties, benefiting 1.43 million IYC annually. After 2018, the program covered 715 poor counties. In 2019, the NIPCPA was incorporated into the basic public health service program, and YYB covered 832 poor counties, with the total number of IYC benefiting reaching 9.47 million [6].

Despite several studies reporting the ability of YYB to reduce anemia [7,8,9], evidence of the effectiveness of YYB in large-scale program settings is limited. It is necessary to systematically evaluate the improvement in the anemia of IYC at scale through NIPCPA. In our study, we aimed to evaluate the effectiveness of an intervention providing YYB on hemoglobin (Hb) and anemia among IYC aged 6–23 mo when delivered through NIPCPA in China.

## 2. Materials and Methods

### 2.1. Description of YYB Intervention in NIPCPA

As infants reached 6 mo of age, their families were informed that they were to receive YYB at the village clinic by village doctors. Each time, the village doctor distributed one box of YYB, which is a month’s supply, and asked about the previous month’s consumption. In some cases, village doctors visited the family at home to distribute YYB. Caregivers were instructed to give one single-dose sachet of YYB daily until children reached 24 mo. Caregivers were given an instructional pamphlet.

YYB was packed in a box of 30 sachets, with 12 g in each sachet. The micronutrient composition and dosage of a single sachet of YYB are shown in Table 1. The formulation was in accordance to the National Food Safety Standard GB/T 22570-2014 [10].

### 2.2. Survey Design and Sampling

This study’s data were obtained from the survey of NIPCPA from 2015 to 2020. Five rounds of surveys were conducted in August–September of 2015, 2017, 2018, 2019 and 2020. All five rounds of surveys had the same design and sampling methods. A stratified and multi-stage probability proportional to size sampling was applied to select IYC aged 6–23 mo in all five surveys, described in detail elsewhere [11,12]. The surveys covered 124 counties in 19 provinces in 2015, 141 counties in 19 provinces in 2017, 148 counties in 19 provinces in 2018, 152 counties in 20 provinces in 2019, and 159 counties in 21 provinces in 2020. IYC who had missing data (i.e., missing sex, age, and Hb items) were excluded from the study.

The purpose and procedures of the study were explained to the parents at enrollment, and only caregivers who gave written informed consent were recruited. The study protocols were approved by the Ethics Committee of the National Institute for Nutrition and Health, Chinese Center for Disease Control and Prevention (No. 2014-001).

### 2.3. Data Collection

#### 2.3.1. Hb

The surveys were conducted by trained staff in the county Maternal and Child Health Hospital (MCH). The selected IYC were invited to the county MCH or township hospital for a physical examination at a specified time. IYCs’ peripheral blood was taken from the ring finger of the left hand and collected with blood tablets. The hemoglobin contents were measured using a portable HemoCue 301 system (HemoCue AB, Ängelholm, Sweden).

#### 2.3.2. Questionnaire Survey

Information on IYCs’ sex, age, birth weight, caregiver’s education and caregiver’s occupation was obtained during interviews with the caregiver of the child. The dietary intake of the IYC was collected by a trained investigator using the 24 h dietary recall approach.

#### 2.3.3. Quality Assurance

The survey scheme and questionnaire were determined after repeated study and discussion by experts. The uniform questionnaires and instruments were provided in all surveys. Investigators were qualified to start field surveys after being trained and evaluated for standardized operations. Specific staff were responsible for questionnaire collection and quality review.

#### 2.3.4. Outcome Measures

The Hb concentrations were adjusted for altitude. Anemia was defined as hemoglobin <110 g/L for IYC aged 6–23 mo according to WHO criteria [13]. Mild, moderate or severe anemia was diagnosed as 100–110, 70–99 g/L or <70 g/L of Hb, respectively.

Complementary foods were divided into eight groups: breast milk; grains, roots, tubers and plantains; pulses, nuts and seeds; dairy products; fresh foods; eggs; vitamin-A-rich fruits and vegetables; and other fruits and vegetables [14].

#### 2.3.5. Statistical Analysis

Data were analyzed with SPSS software (Windows, version 23.0). Means and percentages were used to describe continuous and categorical data, respectively. The data were analyzed and presented in three age groups of 6–11, 12–17, and 18–23 mo. The difference in means among groups was tested by one-way ANOVA. Multiple comparisons were tested with the Bonferroni post hoc test. A non-parametric test was used for the comparison of anemia. For all tests, statistical significance was set at a *p*-value less than 0.05.

Multivariable linear regression was used to assess the estimated effect sizes of YYB, and multivariable logistic regression was performed to estimate the odds ratios (with a 95% CI) of anemia stratified according to age (6–11, 12–17 and 18–23 mo), controlling for sex, race (Han or Minorities), dietary diversity (<5 or ≥5), birth weight, gestational age (<37 weeks or ≥37 weeks), education of caregiver (junior high school or below, high school or above), occupation of caregiver (unemployed or employed) and year of survey (2015, 2017, 2018, 2019 or 2020).

## 3. Results

### 3.1. Characteristics of IYC and Primary Caregivers

Table 2 shows the characteristics of IYC and their caregivers. A total of 36,325, 40,027, 43,831, 44,375 and 46,050 IYC and their caregivers were included in 2015, 2017, 2018, 2019 and 2020, respectively. The mean age of the IYC was approximately 15 mo and almost half were males in each year from 2015 to 2020. Each of the three age groups of 6–11, 12–17 and 18–23 mo old accounted for almost one-third. Almost 75% of the main caregivers were parents. A decreasing percentage of caregivers were educated at junior high school or below from 2015 to 2020 (*p* < 0.001).

### 3.2. Effect of YYB Intervention on Mean Hb

The hemoglobin concentrations of IYC aged 6–23 mo in the NIPCPA from 2015 to 2020 are shown in Table 3. For IYC aged 6–23 mo, there was an overall trend of increase with statistically significant differences from the 2015 to 2020 survey (*p* < 0.001). Compared with the results in the 2015 survey, the hemoglobin concentrations of IYC significantly increased in 2017, 2018, 2019 and 2020. The 2020 survey had the highest increase in mean Hb concentration.

### 3.3. Effect of YYB Intervention on Anemia Prevalence

The anemia prevalence and anemia classifications of IYC aged 6–23 mo in the NIPCPA from 2015 to 2020 are shown in Table 4. The prevalence of anemia appeared to decrease by year. In the 2015 survey, the anemia prevalence was 29.7%. The prevalence of mild, moderate and severe anemia was 18.8%, 10.6% and 0.3%, respectively. In the 2020 survey, the IYC had the highest reduction in the prevalence of anemia, and the proportion of mild and moderate anemia significantly decreased to 12.4% and 5.4%, respectively (*p* < 0.05). Compared with the results in 2015, the anemia rates of IYC aged 6–11, 12–17, 18–23, and 6–23 mo in the 2017, 2018, 2019, and 2020 surveys all showed significant decreases (*p* < 0.05). Compared with the results regarding the anemia rate in 2015, it decreased by 9.4% in 2017, 18.9% in 2018, 28.6% in 2019, and 39.1% in 2020, respectively.

### 3.4. Estimated Quantity of YYB Consumed

Using data on the sachets received and remaining at home, the percentages of IYC according to the quantity of YYB consumed were calculated, as shown in Table 5. There were statistically significant differences in the proportion of IYC using YYB between the yearly observations (*p* < 0.001). From 2015 to 2020, the proportion of YYB with high adherence became higher and the proportion of low adherence became lower in each age group. The percentage of IYC who did not consume YYB significantly decreased from 29.7% to 7.8% among 6–11 mo infants, from 22.1% to 3.9% among 12–17 mo young children, from 18.7% to 3.9% among 18–23 mo young children, respectively.

### 3.5. Multivariable Regression Analyses

The results from this multivariable linear regression analysis, shown in Table 6, reveal that YYB intervention significantly improved Hb concentrations. YYB consumption was significantly associated with a higher mean Hb for children aged 6–11, 12–17 and 18–23 mo when controlling for child’s sex, birth weight, gestational age, dietary diversity, caregiver’s education, caregiver’s occupation and year of survey. In the 12–17 mo age group, the children who consumed more than 90 YYB sachets had a significant increase in Hb concentration compared with those who did not YYB (*p* < 0.001). The results show that the consumption of 270–359 sachets in 12–17 mo IYC was associated with the most significant increase in Hb concentration of 2.189 g/L. In the 18–23 mo age group, the children who consumed more than 180 YYB sachets had a significant increase in Hb concentration (*p* < 0.001). Moreover, the impact of YYB on mean Hb was greater among younger age groups (6–11, 12–17 mo) compared with older children (18–23 mo).

Multivariable logistic regression adjusting for child’s sex, dietary diversity, birth weight, gestational age, caregiver’s education, caregiver’s occupation and year of survey showed that YYB significantly reduced the risk of anemia, stratified by age. For infants aged 6–11 mo, the odds of having anemia were reduced by 17.3% and 20.2% in those consuming less than 90 YYB sachets and 90–179 sachets compared with the infants not consuming YYB, respectively. Furthermore, IYC aged 12–17 mo who consumed more than 90 sachets and IYC aged 18–23 mo who consumed more than 180 sachets had significantly lower odds of anemia compared to those of the same age group who did not consume YYB. The most significant reduction in odds of anemia was observed in the group of IYC aged 12–17 mo who consumed 270 to 359 sachets of YYB, with an almost one-third reduction (OR: 0.671; 95% CI: 0.627, 0.719; *p* < 0.001) (Table 7).

## 4. Discussion

To our knowledge, this is the first systematic nationwide evaluation of the large-scale NIPCPA program of distributing YYB, in terms of its effectiveness in preventing anemia and the dose–response relationship among IYC. This study demonstrates that providing IYC, through NIPCPA, with YYB containing iron and other key micronutrients improved Hb levels and reduced anemia among IYC aged 6–23 mo in China. In the 2015 survey, anemia rates were 29.7% among IYC 6–23 mo of age and even up to 37.8% among infants 6–11 mo of age, which constituted a moderate public health problem. In the 2017 survey, the anemia rate significantly decreased among all age groups. Anemia was present in 26.9% of IYC, which was significantly lower than the national prevalence observed among children (36.9%) during the same period [15]. With the further expansion of the YYB coverage and the increment in adherence to YYB, the anemia rates showed a continuous downward trend, and further reduced to 18.1% among IYC in the 2020 survey, which then constituted a mild public health problem. From 2015 to 2020, mild anemia was predominant among IYC with anemia, followed by moderate anemia. An extremely low percentage of IYC suffered severe anemia. In addition, we also found a statistically significant increment in the concentration of Hb among IYC from the 2015 to 2020 survey.

Many studies have shown that daily or weekly iron supplementation is effective in improving iron nutritional status and further reducing anemia in children. Iron deficiency is not the only cause of nutritional anemia; deficiencies of other micronutrients, such as folic acid, vitamin B_12_ and vitamin A, could also lead to anemia [3,16,17]. Supplementation with multiple micronutrients should be more effective than iron supplementation alone for controlling anemia. Since more than 10 years ago, home fortification or point-of-use fortification interventions have been recognized as an effective measure for the prevention of anemia [18]. The provision of small-quantity lipid-based nutrient supplements decreased the prevalence of anemia by 16% [19]. Home fortification with MNP reduced the risk of anemia by 18%, compared with no intervention or placebo [20]. A study on a pilot Infant and Young Child Nutrition program in the Kyrgyz Republic showed that the prevalence of anemia decreased from 50.6% to 43.8% after one year of MNP intervention [21]. The monitoring results of NIPCPA were consistent with previous studies, indicating that YYB rich in high-quality protein and micronutrients can effectively improve anemia among IYC. In most MNP studies, the dosage of elemental iron was 10–12.5 mg/d and provided primarily as ferrous fumarate. A 2020 Cochrane systematic review reported that MNP with an iron content of 12.5 mg or higher was more effective for reducing anemia and for increasing Hb concentrations compared to MNP with low iron content [20]. The majority of SQ-LNS trials provided only 6–9 mg/d elemental iron and primarily as ferrous sulfate [19]. The recommended nutrient intake for iron was 10 mg/d for infants 6–11 mo of age and 9 mg/d for young children 12–23 mo of age according to Chinese Dietary Reference Intakes (DRIs) [22]. In our study, the iron content in YYB was 7.5 mg/d, in which 2.5 mg iron from NaFeEDTA with a higher absorption rate [23], and another 5 mg of iron in the form of ferric pyrophosphate or ferrous fumarate. The iron dosage used in the current study met the National Food Safety Standard of Complementary Food Supplements (GB 22570-2014) [10], whereas it was lower than the WHO recommended dosage of 10–12.5 mg/d for IYC under two years [24].

YYB was proven to reduce anemia in previous efficacy trials [7,25,26]. A meta-analysis comparing fortification with YYB for IYC between the ages of 6 and 23 mo with no intervention concluded that the average increase in Hb concentration was 0.29 g/dL, with a 60% relative reduction in anemia. When comparing before–after studies, YYB showed a 0.81 g/dL increase in Hb concentration and a 54% relative reduction in anemia [27]. Another meta-analysis of post-only studies with concurrent control determined that YYB was associated with an increase of 4.43 g/L (95% CI:1.55, 7.30) in Hb concentration, and reductions in the prevalence of anemia (risk ratio (RR) = 0.55; 95% CI: 0.45, 0.67) [28].

Despite the well-recognized benefits of supplementation, the effect of implementation has been impeded by poor intake adherence, suggesting that adherence is a key factor in determining the effectiveness of intervention [29,30,31]. In terms of YYB adherence in our study, IYC in each age group represented an increasing proportion in the high-consumption group of YYB and a decreasing proportion in the non- or low-consumption groups from 2015 to 2020. Overall, YYB seems to be increasingly well accepted by IYC and families. After adjusting for potential confounders, multivariable linear regression showed that the intervention with YYB was significantly associated with the increment in Hb concentration. In each age group, the more YYB was consumed, the greater the increase in Hb concentration. In addition, the effect of intervention on the Hb concentration of younger children was greater than that of older children in each consumption group of YYB. The multivariable logistic regression results demonstrate that the intervention was significantly associated with a reduction in anemia. Higher average adherence showed greater reductions in the prevalence of anemia in each age group. Similar to the results of Hb improvement, the results regarding the incidence of anemia suggest that older children aged 18–23 mo who consumed the same amount of YYB did not benefit from YYB to the same extent as younger children did. These results were consistent with those of a previous study [32]. We found that the impact of supplementation with 270 to 359 sachets of YYB for 12–17 mo children was greatest in improving hemoglobin concentration and in reducing anemia prevalence among IYC. The average effect was an increase in Hb concentration by 2.189 g/L and a 32.9 percentage point decrease in the prevalence of anemia. These findings were not in agreement with those of Feng et al., who concluded that YYB significantly improved Hb levels and reduced anemia prevalence during the 1st–9th mo of intervention, but was not effective in the 10th–18th mo of intervention, and the greatest impacts were found at the 7th–9th mo of intervention [9]. The study by Huo et al. showed that Hb was positively influenced by the duration of YYB consumption and the number of sachets per week only for the duration of YYB consumption ≤6 mo, but not >6 mo, by linear regression analysis [33]. The Serdula study found a significant reduction after MNP intervention for one year in the prevalence of anemia among children 12–24 mo of age, but not among children 6–11 mo of age [20]. Overall, the results of our study show that the longer YYB was consumed in accordance with the regular recommended serving size, the better the improvement in Hb concentration and anemia. In addition, the best improvement was observed at the age of 12–17 mo among the same consumption group of YYB. One possible explanation for the effectiveness of YYB not being as great for the age of 18–23 mo as it was for the age of 12–17 mo may be that the capacity to upregulate iron absorption during periods of low iron status may exceed the ability to downregulate iron absorption when iron status is adequate for IYC [34]. The children aged 18–23 mo maybe have a better iron status than those aged 12–17 mo; thus, IYC aged 12–17 mo had greater iron absorption and YYB intervention was more effective.

Here, we present some limits of our study. First, we conducted five rounds of cross-sectional surveys without pre-intervention baseline data or parallel placebo for comparison. In addition, the program’s target population had an age requirement of 6 mo to enter the program and 12 mo to exit the program, making it impossible to conduct follow-up surveys, although the program was ongoing. Second, the collection of data about YYB consumption was based on recalling the number of sachets received and remaining at home by the caregivers, which inevitably resulted in recall bias. Third, it was not recommended to simultaneously consume YYB and other nutrient supplements during the program implementation. Therefore, the questionnaire design did not collect information on the detailed usage of other nutrient supplements apart from YYB. As a result, this study did not fully account for the potential impact of other nutrient supplements on the improvement in anemia.

Our study also has some strengths. We periodically conducted an effectiveness assessment of YYB on anemia in a representative large sample through a large-scale NIPCPA from 2015 to 2020, and made programmatic recommendations, rather than conducting efficacy trials. In addition, the effect of YYB intervention on anemia varied among IYC of different months of age, so this study conducted a group regression analysis to develop our understanding of the dose–response relationship for IYC.

## 5. Conclusions

In conclusion, this study indicates that YYB delivered through the healthcare systems was efficacious in increasing Hb concentrations and reducing the risk of anemia in IYC through the large-scale NIPCPA. It is necessary to analyze the factors that affect the adherence of YYB and continue to promote the consumption of YYB in the NIPCPA.

## Figures and Tables

**Table 1:** Composition of Yingyangbao.

Nutrient	Composition
Energy	220 kJ (52.6 kcal)
Protein	3.0 g
Vitamin A	250 μg RE
Vitamin D	5 μg
Vitamin B_1_	0.5 mg
Vitamin B_2_	0.5 mg
Vitamin B_12_	0.5 μg
Folic acid	75 μg
Calcium	200 mg
Iron	7.5 mg
Zinc	3 mg

**Table 2:** Characteristics of IYC and caregivers enrolled in the NIPCPA survey in China.

Characteristics	2015	2017	2018	2019	2020
*n*	36,325	40,027	43,831	44,375	46,050
Sex					
Male (%)	47.7	48.0	48.4	48.4	48.3
Age (mo), *n*					
6–11	11,659	13,800	13,852	14,881	15,268
12–17	12,578	13,293	14,558	14,224	15,257
18–23	12,088	12,934	15,421	15,270	15,525
Mean age (s.d.) (mo)	15.1 (5.0)	14.9 (5.1)	15.3 (5.1)	15.1 (5.1)	15.1 (5.1)
Preterm birth rate (%)	4.2	4.1	3.8	4.2	4.1
Parents as main caregivers, *n* (%)	75.0	75.6	76.1	75.0	77.7
Caregiver’s education (junior high school or below), *n* (%)	82.9	80.0	77.7	75.8	73.5
Caregiver unemployed, *n* (%)	62.1	65.2	64.1	65.2	63.8

**Table 3:** Concentration of hemoglobin among IYC aged 6–23 mo in the NIPCPA survey from 2015 to 2020 (g/L).

Age Group, mo	2015	2017	2018	2019	2020
6–11	112.2 ± 12.6	113.3 ± 12.1 ^aa^	114.3 ± 12.4 ^aabb^	115.1 ± 11.7 ^aabbcc^	115.9 ± 11.6 ^aabbccdd^
12–17	114.5 ± 13.0	115.3 ± 12.7 ^aa^	116.0 ± 12.5 ^aabb^	116.9 ± 12.1 ^aabbcc^	117.9 ± 11.9 ^aabbccdd^
18–23	118.4 ± 12.4	118.8 ± 12.3	119.1 ± 12.2 ^aa^	120.0 ± 11.9 ^aabbcc^	120.9 ± 11.6 ^aabbccdd^
6–23	115.1 ± 12.9	115.8 ± 12.6 ^aa^	116.6 ± 12.5 ^aabb^	117.4 ± 12.0 ^aabbcc^	118.3 ± 11.9 ^aabbccdd^

^aa^ Compared with 2015, *p* < 0.01. ^bb^ Compared with 2017, *p* < 0.01. ^cc^ Compared with 2018, *p* < 0.01. ^dd^ Compared with 2019, *p* < 0.01.

**Table 4:** Prevalence of anemia among IYC aged 6–23 mo in the NIPCPA survey from 2015 to 2020 (%).

Age Group, mo	Severity of Anemia	2015	2017	2018	2019	2020
6–11	Total	37.8	33.4 ^a^	29.4 ^ab^	26.7 ^abc^	23.1 ^abcd^
	Mild	23.9	21.7 ^a^	18.8 ^ab^	18.2 ^ab^	15.8 ^abcd^
	Moderate	13.6	11.4 ^a^	10.2 ^ab^	8.2 ^abc^	7.0 ^abcd^
	Severe	0.4	0.3	0.4	0.3	0.3
12–17	Total	31.1	28.1 ^a^	25.7 ^ab^	22.2 ^abc^	19.0 ^abcd^
	Mild	19.2	18.0	16.0 ^ab^	14.7 ^abc^	13.1 ^abcd^
	Moderate	11.7	9.8 ^a^	9.5 ^a^	7.2 ^abc^	5.7 ^abcd^
	Severe	0.2	0.3	0.3	0.3	0.3
18–23	Total	20.3	18.6 ^a^	17.8 ^a^	14.9 ^abc^	12.3 ^abcd^
	Mild	13.4	12.3	12.0 ^a^	10.2 ^abc^	8.4 ^abcd^
	Moderate	6.7	6.0	5.6 ^a^	4.4 ^abc^	3.6 ^abcd^
	Severe	0.2	0.2	0.2	0.3	0.3
6–23	Total	29.7	26.9 ^a^	24.1 ^ab^	21.2 ^abc^	18.1 ^abcd^
	Mild	18.8	17.4 ^a^	15.5 ^ab^	14.3 ^abc^	12.4 ^abcd^
	Moderate	10.6	9.1 ^a^	8.3 ^ab^	6.6 ^abc^	5.4 ^abcd^
	Severe	0.3	0.3	0.3	0.3	0.3

^a^ Compared with 2015, *p* < 0.05. ^b^ Compared with 2017, *p* < 0.05. ^c^ Compared with 2018, *p* < 0.05. ^d^ Compared with 2019, *p* < 0.05.

**Table 5:** Proportion of the consumption of YYB among IYC aged 6–23 mo in the NIPCPA survey from 2015 to 2020 (%).

Age Group, mo	YYB Consumed, Sachets	2015	2017	2018	2019	2020
6–11	0	29.7	18.6 ^a^	12.7 ^ab^	10.2 ^abc^	7.8 ^abcd^
	<90	49.2	54.7 ^a^	57.6 ^ab^	60.0 ^abc^	59.4 ^abc^
	90–179	21.2	26.8 ^a^	29.7 ^ab^	29.8 ^ab^	32.8 ^abcd^
12–17	0	22.1	14.8 ^a^	8.4 ^ab^	5.0 ^abc^	3.9 ^abcd^
	<90	18.9	13.2 ^a^	12.8 ^a^	12.1 ^ab^	12.3 ^a^
	90–179	24.5	24.7	27.5 ^ab^	28.3 ^ab^	28.8 ^ab^
	180–269	22.4	30.8 ^a^	32.3 ^a^	34.6 ^abc^	34.8 ^abc^
	270–359	12.2	16.4 ^a^	19.0 ^ab^	20.0 ^ab^	20.1 ^ab^
18–23	0	18.7	11.8 ^a^	7.9 ^ab^	4.6 ^abc^	3.9 ^abcd^
	<90	12.7	7.9 ^a^	8.2 ^a^	6.5 ^abc^	6.9 ^abc^
	90–179	12.8	9.2 ^a^	9.8 ^a^	9.2 ^a^	9.8 ^a^
	180–269	10.3	9.2 ^a^	9.5	9.3 ^a^	9.6
	270–359	22.9	20.7 ^a^	22.7 ^b^	23.4 ^b^	23.5 ^b^
	360–449	16.8	26.5 ^a^	26.7 ^a^	30.2 ^abc^	30.1 ^abc^
	450–539	5.7	14.7 ^a^	15.3 ^a^	16.8 ^abc^	16.2 ^ab^

^a^ Compared with 2015, *p* < 0.05. ^b^ Compared with 2017, *p* < 0.05. ^c^ Compared with 2018, *p* < 0.05. ^d^ Compared with 2019, *p* < 0.05.

**Table 6:** Determinants of hemoglobin concentrations among IYC aged 6–23 mo in the NIPCPA survey from 2015 to 2020 ^1^.

Dependent Variable	6–11 mo	12–17 mo	18–23 mo
Sex (reference group = male)	0.440 ** (0.092)	0.316 ** (0.095)	0.131 (0.091)
YYB consumed, sachets (reference group = 0)			
<90	0.696 ** (0.136)	0.218 (0.195)	−0.337 (0.219)
90–179	0.974 ** (0.150)	1.122 ** (0.175)	0.364 (0.210)
180–269		1.295 ** (0.173)	1.202 ** (0.213)
270–359		2.189 ** (0.188)	0.864 ** (0.181)
360–449			1.212 ** (0.179)
450–539			1.644 ** (0.198)
Dietary diversity (reference group < 5)	0.585 ** (0.095)	1.156 ** (0.096)	1.418 ** (0.094)
Survey time (reference group = 2015)			
2017	1.047 ** (0.153)	0.653 ** (0.156)	0.121 (0.154)
2018	1.949 ** (0.154)	1.113 ** (0.154)	0.322 * (0.149)
2019	2.597 ** (0.152)	1.927 ** (0.156)	0.962 ** (0.151)
2020	3.450 ** (0.152)	2.899 ** (0.154)	1.793 ** (0.151)

^1^ Values are the regression coefficient (SE) obtained from multivariable linear regression analyses adjusted for variables listed in the table and variables for birth weight, gestational age, caregiver’s education and caregiver’s occupation, which are not listed. ** *p* < 0.01, * *p* < 0.05.

**Table 7:** Determinants of anemia among IYC aged 6–23 mo in the NIPCPA survey from 2015 to 2020 ^1^.

Dependent Variable	6–11 mo OR (95% CI)	12–17 mo OR (95% CI)	18–23 mo OR (95% CI)
Sex (reference group = male)	0.911 ** (0.881, 0.942)	0.912 ** (0.881, 0.945)	0.940 ** (0.903, 0.979)
Dietary diversity	0.936 ** (0.904, 0.969)	0.842 ** (0.813, 0.872)	0.771 ** (0.739, 0.803)
YYB consumed, sachets (reference group = 0)			
<90	0.827 ** (0.789, 0.867)	0.989 (0.924, 1.059)	1.105 * (1.009, 1.209)
90–179	0.798 ** (0.756, 0.841)	0.843 ** (0.792, 0.897)	0.971 (0.889, 1.060)
180–269		0.799 ** (0.751, 0.849)	0.863 ** (0.788, 0.945)
270–359		0.671 ** (0.627, 0.719)	0.827 ** (0.766, 0.894)
360–449			0.770 ** (0.713, 0.831)
450–539			0.755 ** (0.693, 0.823)
Survey time (reference group = 2015)			
2017	0.839 ** (0.796, 0.884)	0.896 ** (0.848, 0.946)	0.947 (0.888, 1.010)
2018	0.708 ** (0.671, 0.747)	0.817 ** (0.774, 0.863)	0.913 ** (0.858, 0.973)
2019	0.632 ** (0.599, 0.666)	0.690 ** (0.652, 0.730)	0.770 ** (0.721, 0.822)
2020	0.525 ** (0.497, 0.555)	0.570 ** (0.538, 0.603)	0.619 ** (0.579, 0.663)

OR, odds ratio; CI, confidence interval. ^1^ Values are OR (95% CI) obtained from multivariable logistic regression analyses adjusted for variables listed in the table and variables for birth weight, gestational age, caregiver’s education and caregiver’s occupation, which are not listed. ** *p* < 0.01, * *p* < 0.05.

## Data Availability

Not applicable.
